# Modulation of lipopolysaccharide-induced neuronal response by activation of the enteric nervous system

**DOI:** 10.1186/s12974-014-0202-7

**Published:** 2014-12-12

**Authors:** Sabrina Coquenlorge, Emilie Duchalais, Julien Chevalier, Francois Cossais, Malvyne Rolli-Derkinderen, Michel Neunlist

**Affiliations:** Neuropathies of the enteric nervous system and digestive diseases, INSERM UMR913, School of Medicine, University of Nantes, 1, rue Gaston Veil, Nantes, F-44035 France; University of Nantes, 1 quai de Tourville, BP 13522, Nantes, Cedex 1 F-44035 France; Institut des Maladies de l’Appareil Digestif, Centre Hospitalier Universitaire, Nantes, Hopital Hôtel-Dieu, 1 place Alexis Ricordeau, Nantes, F-44093 France; Centre de Recherche en Nutrition Humaine, Hopital Hôtel-Dieu, 1 place Alexis Ricordeau, Nantes, F-44093 France

**Keywords:** Enteric nervous system, LPS, TNF-α, AMPK, ERK

## Abstract

**Background:**

Evidence continues to mount concerning the importance of the enteric nervous system (ENS) in controlling numerous intestinal functions in addition to motility and epithelial functions. Nevertheless, little is known concerning the direct participation of the ENS in the inflammatory response of the gut during infectious or inflammatory insults. In the present study we analyzed the ENS response to bacterial lipopolysaccharide, in particular the production of a major proinflammatory cytokine, tumor necrosis factor-alpha (TNF-α).

**Methods:**

TNF-α expression (measured by qPCR, quantitative Polymerase Chain Reaction) and production (measured by ELISA) were measured in human longitudinal muscle-myenteric plexus (LMMP) and rat ENS primary cultures (rENSpc). They were either treated or not treated with lipopolysaccharide (LPS) in the presence or not of electrical field stimulation (EFS). Activation of extracellular signal-regulated kinase (ERK) and 5’-adenosine monophosphate-activated protein kinase (AMPK) pathways was analyzed by immunocytochemistry and Western blot analysis. Their implications were studied using specific inhibitors (U0126, mitogen-activated protein kinase kinase, MEK, inhibitor and C compound, AMPK inhibitor). We also analyzed toll-like receptor 2 (TLR2) expression and interleukin-6 (IL-6) production after LPS treatment simultaneously with EFS or TNF-α-neutralizing antibody.

**Results:**

Treatment of human LMMP or rENSpc with LPS induced an increase in TNF-α production. Activation of the ENS by EFS significantly inhibited TNF-α production. This regulation occurred at the transcriptional level. Signaling analyses showed that LPS induced activation of ERK but not AMPK, which was constitutively activated in rENSpc neurons. Both U0126 and C compound almost completely prevented LPS-induced TNF-α production. In the presence of LPS, EFS inhibited the ERK and AMPK pathways. In addition, we demonstrated using TNF-α-neutralizing antibody that LPS-induced TNF-α production increased TLR2 expression and reduced IL-6 production.

**Conclusions:**

Our results show that LPS induced TNF-α production by enteric neurons through activation of the canonical ERK pathway and also in an AMPK-dependent manner. ENS activation through the inhibition of these pathways decreased TNF-α production, thereby modulating the inflammatory response induced by endotoxin.

**Electronic supplementary material:**

The online version of this article (doi:10.1186/s12974-014-0202-7) contains supplementary material, which is available to authorized users.

## Background

The enteric nervous system (ENS), composed of neurons and enteric glial cells (EGC), is a central regulator of gastrointestinal functions encompassing gut motility, electrolyte transport and intestinal epithelial barrier (IEB) functions [1]. Recently, the ENS has been recognized as a major player in gut protection in response to pathogen or inflammatory insult [2]. Conversely, the ENS is also affected in disease, in particular in inflammatory bowel diseases (IBD). Alterations in ENS functions and phenotype (altered excitability and neuroplastic changes) occur in IBD [3,4]. These changes are associated with gastrointestinal (GI) dysfunctions such as altered motility, diarrhea and even pain. The ENS could also be directly involved in the inflammatory response to infectious or inflammatory challenges. Indeed, in animal models of colitis, enteric neuronal hyper- and hypoplasia was associated with increased and reduced production of TNF-α, respectively [5]. In addition, hypertrophy and hyperplasia of enteric neurons has been reported in IBD [6]. However, the mechanism responsible for neuronal modulation of the severity of inflammation remains unknown. Being putatively due to the modulation of neuroimmune interactions, it is tempting to speculate that enteric neurons could directly produce and regulate key cytokines involved in IBD.

During inflammation, the ENS responds to a wide range of mediators, such as cytokines [7]. The ENS can also respond to bacterial challenges such as lipopolysaccharide, as it expresses a wide array of toll-like receptors (TLRs). Enteric neurons express TLR4 [8,9]. TLR3 and TLR7 are expressed in the myenteric and submucosal plexi. TLR3, TLR4 and TLR7 are also expressed in EGC [10-13]. The functional consequences of the activation of TLRs by their ligands in enteric neurons remain largely unknown. TLR2 was nevertheless shown to be necessary for maintaining ENS integrity and protection from colitis [14]. Stimulation of TLR4 in EGC induced the release of nitric oxide [12]. However, the ability of enteric neurons to respond to lipopolysaccharides (LPS) and to synthesize cytokines such as TNF-α, as well as the signaling pathways involved, remain unknown.

The overall aim of this study was to determine whether the ENS can directly respond to LPS by producing the major proinflammatory cytokine TNF-α, and whether ENS activity can modulate this production.

## Methods

### Generation of enteric nervous system cultures

Rat ENS primary cultures (rENSpc) were performed as previously described using the small intestines of E15 Sprague-Dawley rat embryos (Janvier Laboratories SA, Le Genest-St-Isle, France) [15]. These procedures were approved by the local Animal Care and Use Committee (agreement E. 44011; INSERM, Nantes, France). Briefly, the small intestines of rat embryos were removed, diced in Hank’s Buffered Salt Solution (Sigma-Aldrich, Saint-Louis, Missouri, United States) and collected in 5 mL of Dulbecco’s modified Eagle’s medium (DMEM)-F12 (Gibco®, Life Technologies, Carlsbad, California, United States) (1:1) for digestion at 37°C for 15 minutes in 0.1% trypsin (Sigma-Aldrich). The trypsin reaction was stopped by adding medium containing 10% fetal calf serum and then treated by DNase I 0.01% (Sigma-Aldrich) for 10 minutes at 37°C. After triturating with a 10 mL pipette, cells were centrifuged at 750 rpm for 10 minutes. Cells were counted and then seeded at a density of 2.4 × 10^5^ cells/cm^−2^ on 24-well plates previously coated with a solution of gelatin (0.5%; Sigma-Aldrich) in sterile phosphate buffered saline. After 24 hours, the medium was replaced with a serum-free medium DMEM-F12 (1:1) containing 1% of N-2 supplement (Life Technologies. Cultures were maintained for 14 days. Using this method, rENSpc is composed of well-organized ganglia interconnected by interganglionic fiber strands forming a network that lays on a smooth muscle cells monolayer (smooth muscle actin or SMA positive cells). Fluorescence-activated cell sorting (FACS) analysis, aimed to reveal the cellular composition of the rENSpc, revealed that enteric neurons and glial cells represent 5 to 6% and 20 to 40%, respectively, of cells present in culture (personal observation). Half of the medium was replaced every other day and the day before the experiment.

### Enteric nervous system treatments

**Figure 1:**
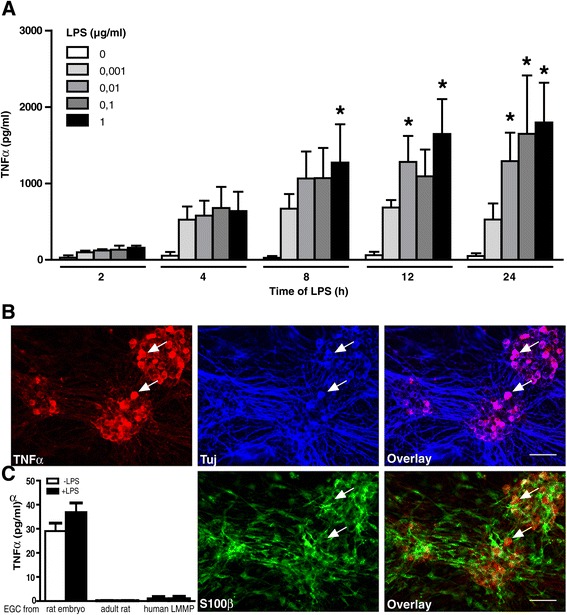
**Enteric neurons produce TNF-α in response to LPS stimulation.** **(A)** rENSpc were treated in a time- and dose-dependent manner with LPS. Quantification of TNF-α secretion was measured by ELISA. Values represent the mean ± SEM of between three and seven independent samples per condition (two-way ANOVA test followed by a Bonferroni *post-hoc* test; *P <0.05 as compared with the same time point without LPS). **(B)** Representative images of TNF-α localization in the rENSpc (four independent samples). Immunocytochemical triple labeling of ENS cultures were performed using anti-TNF-α, anti-S100β (glial marker) and anti-Tuj (neuronal marker) antibodies. Examples of neurons expressing TNF-α are depicted with white arrowheads. Scale bar: 50 μm. **(C)** LPS treatment of enteric glial cell cultures did not induce TNF-α production (between four and ten independent experiments). EGC, enteric glial cells; LMMP, longitudinal muscle/myenteric plexus; LPS, lipopolysaccharides; rENSpc, rat enteric nervous system primary culture; S100β, S100 calcium binding protein beta; SEM, standard error of the mean; TNF-α, tumor necrosis factor alpha; Tuj, βIII-tubulin.

The rENSpc were treated with lipopolysaccharides (*Escherichia coli and Salmonella typhosa;* 1:1, Sigma-Aldrich) at 0.1 μg/ml for the indicated time, except for Figure 1A, where different concentrations were tested. For the purpose of establishing which pathways and receptors are implicated in TNF-α and TLR2 regulation, U0126 (mitogen-activated protein kinase kinase 1/2 or MEK1/2 inhibitor; 10 μM), compound C (5’-adenosine monophosphate-activated protein kinase (AMPK) inhibitor; 10 μM) (Calbiochem, Merk Millipore, Billerica, Massachusetts, United States) and anti-rat TNF-α (1 and 10 μg/ml; eBiosciences, San Diego, California, United States) were added 30 minutes prior to the addition of LPS or ENS stimulation. Pam3CSK4 (TLR1/2 agonist; 100 ng/ml; Invivogen, San Diego, California, United States), A438079 (selective P2X_7_ antagonist; 30 μM; Tocris Bioscience, Bristol, United Kingdom), adenosine-5'-triphosphate (ATP) (100 μM) and 2’(3’)-O-(4-benzoylbenzoyl) adenosine-5'-triphosphate triethylammonium salt (BzATP) (selective P2X7 agonist; 100 μM; Sigma-Aldrich) were also used to treat ENS plus or minus LPS.

### Enteric nervous system activation

To study the effect of neuronal activity on cytokine secretion, rENSpc were electrically stimulated in 24-well plates fitted with a pair of platinum electrodes connected to an electrical stimulator (DualImpedance Research Stimulator, Harvard Apparatus Ltd, Edenbridge, United Kingdom). The electrical field stimulation (EFS) parameters used were trains of constant current pulses (pulse duration: 20 μs; amplitude: 8 V; frequency: 15 Hz) applied for seven hours, with reversal of electrode polarity every 30 minutes, and supernatants and lysates were collected after 24 hours for ELISA TNF-α measurements and quantitative PCR (qPCR). Neuronal activation was verified by analysis of *c-Fos* expression after seven hours of EFS (Additional file 1). Putative neuronal damage induced by EFS was also verified. Following seven hours of EFS, no change in neuron-specific enolase (NSE) in the culture medium or in protein gene product (PGP) 9.5 expression was observed as compared to control (non-stimulated condition), suggesting that EFS did not affect neuronal viability. Neuronal activity during the seven hours was also measured using an essential neuromediator, ATP.

### Enteric glial cells

Primary cultures of human and adult rat EGCs were obtained according to the procedure described by Soret *et al*. [16]. Briefly, human EGC were originated from colonic resections of patients with colorectal cancer (according to the guidelines of the French Ethics Committee for Research on Human and registered under the number DC-2008-402). Adult rat EGC were obtained from entire small intestines of Sprague-Dawley rats. After dissection of rat or human segments, the remaining longitudinal muscle-myenteric plexus (LMMP) was placed in GentleMACS tubes C in a GentleMACS dissociator (MiltenyiBiotec, Bergisch Gladbach, Germany) for enzymatic digestion (250 μl protease, 5 g/l^−1^; 250 μl collagenase, 20 g/l^−1^; 400 μl bovine serum albumin, 50 g/l^−1^; Sigma-Aldrich) and mechanical dissociation in DMEM/F12 medium (supplemented with 10% heat-inactivated fetal calf serum, 100 IU/ml penicillin, 100 μg/ml streptomycin, 1.1 μg/ml amphotericin B, 20 μg/ml gentamicin, 6 mM glutamine and 2.1 g/l NaHCO_3_; Life Technologies). Following gentle agitation at 37°C, pellets were washed and resuspended in DMEM/F12 complete medium. The suspension was placed under a microscope and ganglia were picked up using a pipette with a sterile tip and seeded in 24-well plate coated with gelatin. After 48 hours, the medium was entirely replaced and only EGC proliferated to reach confluency after two to four weeks. The primary cultures of EGC from rat embryos (JUG2 cell line) was obtained as described by Bach-Ngohou *et al*. [17] and derived from ENS primary culture after trypsinization and differential centrifugation. EGC were treated with LPS at a concentration of 0.1 μg/ml for seven hours before collecting supernatants.

### Human tissue experiments

Colonic segments were pinned in a dissection dish containing ice-cold, sterile oxygenated Krebs solution (Sigma-Aldrich). Mucosae, submucosal and circular muscles were carefully removed under a dissection microscope and the remaining LMMP was placed in a 24-well plate containing DMEM/F12 complete medium (supplemented as in EGC primary culture procedure). The following day, the medium was replaced with 1:1 DMEM-F12 containing 1% N-2 supplement, and specimens were electrically stimulated with the same equipment and parameters as for rENSpc stimulation. After seven hours, supernatants and lysates were collected and tissues were weighted to normalize the ELISA analysis.

### Immunofluorescence

After treatments, rENSpc were fixed in PBS containing 4% paraformaldehyde at room temperature for 30 minutes. Following pre-incubation in PBS containing 5% donkey serum and 0.5% Triton™ X-100 (Sigma-Aldrich) for 30 minutes, cells were incubated with the following primary antibodies overnight at 4°C: rabbit anti-phospho-p44/p42 (P-ERK), rabbit anti-phospho-acetyl coenzyme A (CoA) carboxylase (P-ACC; 1:500; Cell Signaling Technology, Beverly, Massachusetts, United States), goat anti-TLR2 (1:50; Santa Cruz Biotechnology, Dallas, Texas, United States), and goat anti-TNF-α (1:200; Santa Cruz Biotechnology). Anti-rabbit and anti-goat conjugated to Cyanine3 (1:500; Jackson Immunoresearch, West Grove, Pennsylvania, United States) were used as secondary antibodies for one hour. In the following step, the cells were labelled with primary antibodies for neuronal or glial markers: mouse anti-Hu (1:200; Molecular Probes®, Life Technologies), rabbit anti-PGP9.5 (1:1,000; Ultraclone Limited, Tebu-bio, Le Perray-en-Yvelines, France), mouse anti-Sox10 (1:500; R&D systems, Minneapolis, Minnesota, United States), rabbit anti-S100b (1:1,000; DakoCytomation, Glostrup, Denmark), mouse anti-S100b (1:1,000, Abcam, Cambridge, United Kingdom), and mouse anti-β-tubulin III (1:1,000; Sigma-Aldrich). After secondary antibodies (FluoProbes™488; 1:200; Interchim or Cyanine5; 1:200; Jackson Immunoresearch), images were acquired with an Olympus IX 50 fluorescence microscope coupled to a digital camera (model DP71, Olympus), analyzed with Cell B software (Soft Imaging System, Olympus), and treated with Image J software (National Institute of Health, Bethesda, Maryland, United States).

### Immunoblotting

ENS cells cultured for different times (as indicated), with or without stimuli, LPS or agonists, were lysed in RA1 (Nucleospin RNAII, Macherey-Nagel, Düren, Germany) to separate RNA and proteins. Proteins were precipitated and pellets were resuspended in Phosphate-buffered saline/tris (2-carboxyethyl)phosphine (PSB/TCEP) (Macherey-Nagel). Samples were processed for electrophoresis using NuPAGE™ MES SDS buffer kit (Life Technologies) and separated on 4 to 12% Bis-Tris gels (NuPAGE, Life Technologies). Proteins were transferred to nitrocellulose membranes with the iBlot™ system (Life Technologies). After blocking with Tris-buffered saline (TBS), 0.1% Tween 20 and 5% nonfat dry milk for one hour, blots were incubated overnight at 4°C with primary antibodies diluted in TBS and 5% nonfat dry milk for rabbit anti-phospho-Thr172-AMPK (P-AMPK), rabbit anti-AMPK, rabbit anti-phospho-p44/p42 (P-ERK), rabbit anti-p44/p42 and rabbit anti-phospho-ACC (1:500; Cell Signaling Technology), and mouse anti-beta-actin (1:5000; Sigma-Aldrich). Immunoblots were probed with the appropriate horseradish peroxidase-conjugated secondary antibodies (Life Technologies) and visualized by chemiluminescence (ECL Crescendo or Forte, Merk Millipore). Quantitative analysis was performed using Image J software. The value of phosphorylated and total protein immunoreactivity was normalized to the amount of β-actin immunoreactivity and expressed as the fold increase relative to the control, taken as 1.

### Real-time quantitative PCR analysis

According to the manufacturer’s recommendations, total RNA extraction from cells was performed with Nucleospin RNAII kit (Macherey-Nagel) and 1 μg purified RNA was denatured and processed for reverse transcription using Superscript II reverse transcriptase (Life Technologies). PCR amplifications were performed using the Absolute Blue SYBR green fluorescein kit (Roche, Basel, Schweiz) run on a Rotor-Gene (Qiagen, Venlo, The Netherlands). The following primers were used:

S6 number: [GenBank:NM_001010]. Forward primer: 5’-CCAAGCTTATTCAGCGTCTTGTTACTCC-3’. Reverse primer: 5’-CCCTCGAGTCCTTCATTCTCTTGGC-3’. TNF-α number: [GenBank:NM_012675.3]. Forward primer: 5’-CTCATTCCTGCTCGTGGCGGG-3’. Reverse primer: 5’-TACGACGTGGGCTACGGGCTT-3’.TLR2 number: [GenBank:NM_198769.2]. Forward primer: 5’-CAGATGGCCAGAGGACTCA-3’. Reverse primer: 5’-AATGGCCTTCCCTTGAGAG-3’.

Relative quantification of gene expression was determined using the standard curve method and endogenous control ribosomal protein S6 mRNA. The ratios, TNF-α/S6 or TLR2/S6, were compared with control experimental conditions normalized to 1.

### ELISA

The concentrations of TNF-α and IL-6 were determined in rENSpc and human LMMP specimen culture supernatants by ELISA (BD OptEIA™ ELISA Set, BD Biosciences, Franklin Lakes, New Jersey, United States) according to the manufacturer's instructions. Absorbance measurements were performed at 450 nm on a spectrophotometric enzyme-linked immunosorbent sandwich assay (ELISA) plate reader (Varioskan™, Thermo Scientific, Waltham, Massachusetts, United States) using the SkanIt software (Thermo Scientific). For human LMMP specimens, the TNF-α concentration was expressed related to the weight of the specimen.

### Statistical analysis

Data resulted from independent experiments performed in duplicate or triplicate. Statistical significance was evaluated using GraphPad Prism software (GraphPad Software Inc., La Jolla, California, United States). For time- and dose-dependent experiments, a two-way ANOVA test followed by a Bonferroni *post-hoc* test was used. The time-dependent response to LPS in Western blots was analyzed with a Kruskal-Wallis nonparametric ANOVA test, followed by Dunn’s *post-hoc* test. The differences between groups were calculated by a two-tailed Student’s t-test for nonparametric and unpaired data, or by Mann-Whitney U test.

## Results

### Neurons of the enteric nervous system produce TNF-α in response to lipopolysaccharide stimulation

To determine whether the ENS could respond to LPS by producing proinflammatory cytokines, we measured TNF-α concentrations in the supernatants of rENSpc treated with different concentrations of LPS for two to 24 hours. We have verified that neurons of the rENSpc expressed the TLR4 and were thereby able to respond to LPS (Additional file 2). The TNF-α concentration was significantly increased after eight hours of 1 μg/ml LPS stimulation, and between 12 to 24 hours for LPS concentrations ranging from 0.01 to 1 μg/ml (Figure 1A). We chose the intermediate LPS concentration (0.1 μg/ml) for the rest of the study. Using immunocytochemistry, we further aimed to identify the ENS cell type (neuronal or glial) expressing TNF-α. In rENSpc, enteric neurons were organized in ganglia connected to each other by interganglionic fiber strands as demonstrated by immunostaining using βIII-tubulin (Tuj) antibody (Figure 1B). EGCs, identified by S100β immunostaining, were also present in enteric ganglia (Figure 1B). We showed that in rENSpc treated with LPS for 24 hours, TNF-α immunoreactivity was detected in the cytoplasm of Tuj-positive cells, but not in S100β-positive cells (Figure 1B). To confirm that EGC do not produce TNF-α, we measured TNF-α production from human, adult and embryonic rat EGC in response to treatment with LPS. None of these cells responded to LPS treatment with an increase in TNF-α production (Figure 1C). Our results show that enteric neurons are the cells of the rENSpc producing TNF-α in response to LPS stimulation.

### Rat enteric nervous system activation inhibits the lipopolysaccharide-induced increase in TNF-α transcript and protein levels

**Figure 2:**
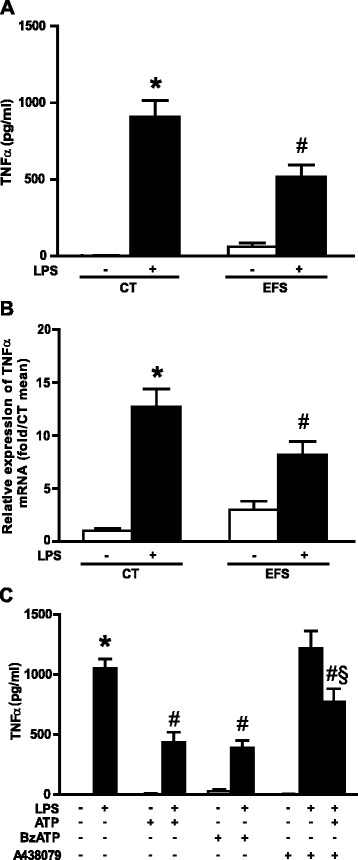
**ENS activation in rat primary cultures inhibits the LPS-induced increase in TNF-α transcript and protein levels.** **(A)** Evaluation of the impact of electrical field stimulation (EFS) on TNF-α secretion was performed by ELISA in rENSpc treated (+) or not (−) with LPS (0.1 μg/ml) (twelve independent experiments). **(B)** *TNF-α* mRNA expression was measured in rENSpc by qPCR (twelve independent experiments). **(C)** The effect of ATP (100 μM) and involvement of P2X7 receptor on TNF-α secretion were measured by ELISA in ENS cultures pretreated or not with BzATP (P2X7 agonist; 100 μM) or A439079 (P2X7 antagonist; 30 μM) (five independent experiments). Values represent the mean ± SEM (Mann-Whitney U test; *P <0.05 as compared with control without LPS; #*P* <0.05 LPS with EFS, agonist or antagonist versus LPS alone; §*P* <0.05 LPS with ATP compared with LPS with ATP in presence of A438079). ATP, adenosine-5'-triphosphate; BzATP, 2’(3’)-O-(4-benzoylbenzoyl) adenosine-5'-triphosphate triethylammonium salt; CT, control; EFS, electrical field stimulation; LPS, lipopolysaccharide; mRNA, messenger ribonucleic acid; rENSpc, rat enteric nervous system primary culture; SEM, standard error of the mean; TNF-α, tumor necrosis factor alpha.

To assess if ENS activity could regulate TNF-α production, we measured LPS-induced TNF-α production in the supernatants of rENSpc after EFS and in those not treated with EFS. EFS had no significant effect on basal TNF-α production. However, EFS significantly inhibited TNF-α production induced by LPS in rENSpc (Figure 2A). qPCR analysis indicated that EFS inhibited the increase in *TNF-α* mRNA level observed after LPS (Figure 2B). These results show that LPS induce an increase in TNF-α transcript and protein levels, and that ENS activation reduces TNF-α production.

### ATP inhibits lipopolysaccharide-induced production of TNF-α through P2X7 receptor activation

Various studies have demonstrated the anti-inflammatory effects of adenosine receptor activation [18,19] and ATP is a common intercellular messenger produced by enteric neurons [20]. To determine their putative regulatory role in LPS-induced TNF-α production by the ENS, we studied the potential role of ATP and purinergic receptor activation. An increased extracellular ATP concentration remarkably decreased the LPS-induced production of TNF-α. The application of BzATP, a P2X7 agonist, induced a similar reduction in TNF-α production, as did ATP. The ATP effects were partially reversed by the selective P2X7 antagonist, A438079 (Figure 2C). These results show that LPS-induced TNF-α production is reduced by ATP stimulation of P2X7 receptors.

### ERK and AMPK inhibition prevent lipopolysaccharide-induced TNF-α upregulation

**Figure 3:**
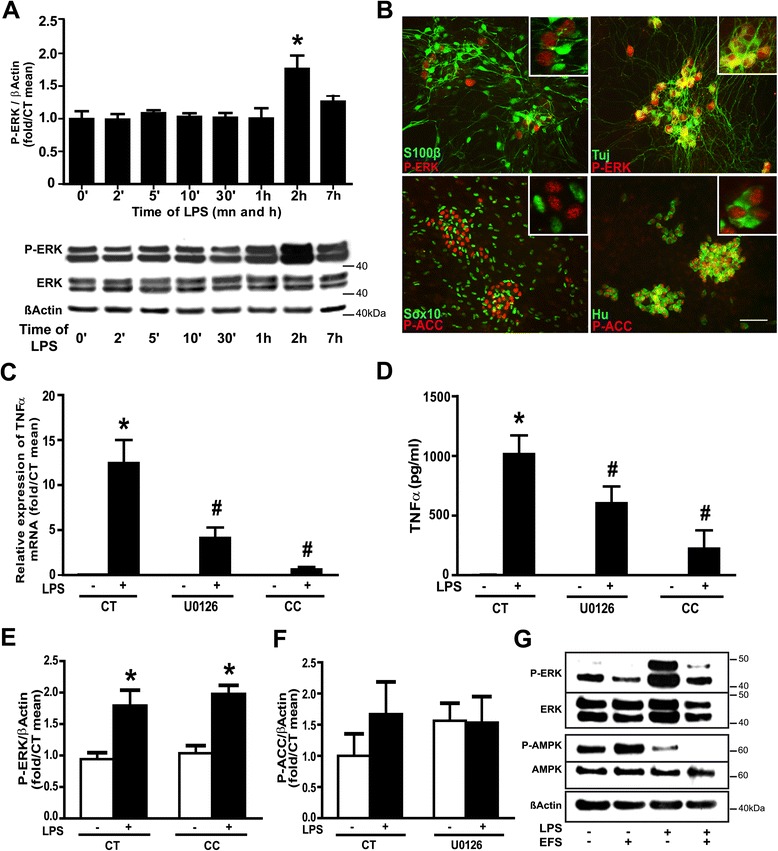
**ERK and AMPK inhibition prevent LPS-induced increase in TNF-α transcript and protein levels.** **(A)** ERK pathway activation in rENSpc treated with LPS from two minutes to seven hours was measured by Western blotting using phospho-ERK antibodies (P-ERK). The relative amount of P-ERK was determined by normalizing with β-actin (four to eight independent experiments). Values represent the mean ± SEM as fold/mean t0 (one-way ANOVA test followed by Dunn’s *post-hoc* test; *P <0.05 as compared with t0 without LPS). **(B)** Localization of activated − ERK and − AMPK in rENSpc treated for two hours with LPS was performed by immunocytochemistry using P-ERK or phospho-Acetyl-CoA Carboxylase (P-ACC), with S100β and Sox10 (glial markers) or Hu and Tuj (βIII-tubulin, neuronal markers). Scale bar: 50 μm. Inserts show higher magnification images (40x for P-ERK and 100x for P-ACC). ERK- and AMPK-dependent pathway contribution to TNFα production were assessed using MEK1/2 inhibitor (U0126; 10 μM) and AMPK inhibitor (C compound, CC; 10 μM) co-treatment on rENSpc treated (+) or not (−) with LPS for seven hours. **(C)** Quantification of TNF-α mRNA by qPCR (six to 11 independent samples). **(D)** TNF-α secretion was assayed by ELISA with or without the presence of inhibitors (six independent samples). The independence of the ERK and AMPK pathways was determined by Western blotting. **(E)** ERK phosphorylation was not affected by CC and **(F)** ACC phosphorylation was not modified by U0126 (eight and three independent experiments, respectively). (Mann-Whitney U test; *P <0.05 as compared with control without LPS; #*P* <0.05 as compared with LPS without inhibitor). **(G)** The EFS impact on ERK and AMPK activation was measured on rENSpc treated (+) or not (−) with LPS and EFS for two hours by Western blotting using P-ERK and P-AMPK antibodies. Immunoblots are representative of five independent experiments with similar results.

To decipher the signaling pathways responsible for LPS-induced TNF-α synthesis by the ENS, and its modulation by EFS, we analyzed the possible implication of the canonical extracellular signal-regulated kinase (ERK) pathway, and also of the 5'-adenosine monophosphate-activated protein kinase (AMPK) pathway. By Western blot analysis using phospho-specific antibodies recognizing the active form of the ERKs, we showed that phosphorylation of ERKs was increased after two hours of LPS stimulation, with no change in ERK expression (Figure 3A). Activation of the AMPK pathway can be measured by the phosphorylation of the Thr172 of the AMPK itself, or by the phosphorylation of acetyl-CoA carboxylase (ACC), an AMPK target, on its Ser19. Neither AMPK nor ACC phosphorylation changed significantly over time, with or without LPS treatment (data not shown). Immunocytochemical analyses revealed that the immunoreactivity for phospho-ERK and phospho-ACC concurred with Tuj and Hu (neuronal markers), but not with S100β or Sox10 (glial markers) immunoreactivity (Figure 3B). These data show that, while the AMPK pathway was constitutively activated in neuronal cells, ERKs were activated in neurons after LPS treatment. Pretreatment of rENSpc with U0126 or compound C inhibitors of the ERK or AMPK pathways, respectively, significantly reduced *TNF-α* mRNA expression (Figure 3C) and TNF-α production (Figure 3D). Interestingly, ERK phosphorylation was not affected by compound C (Figure 3E), and ACC-phosphorylation was not modified by U0126 (Figure 3F), again suggesting the independence of the two pathways. These results demonstrate that the AMPK and ERK pathways participate independently in LPS induction of TNF-α production by the ENS.

### Electrical field stimulation inhibits AMPK and lipopolysaccharide-induced ERK activation

To determine if EFS could inhibit TNF-α production by modulating the ERK and AMPK pathways, we analyzed ERK and AMPK activation in rENSpc treated, or not, with LPS, in the presence or absence of EFS. EFS had no significant effect on basal pathway activity but, combined with LPS treatment, it significantly reduced the phosphorylation of ERK and AMPK compared to the LPS conditions (Figure 3G). This suggests that ENS activation limits TNF-α production by inhibition of LPS-induced ERK activation and decrease of AMPK activity.

### Human enteric nervous system activation inhibits the lipopolysaccharide-induced increase in TNF-α production

**Figure 4:**
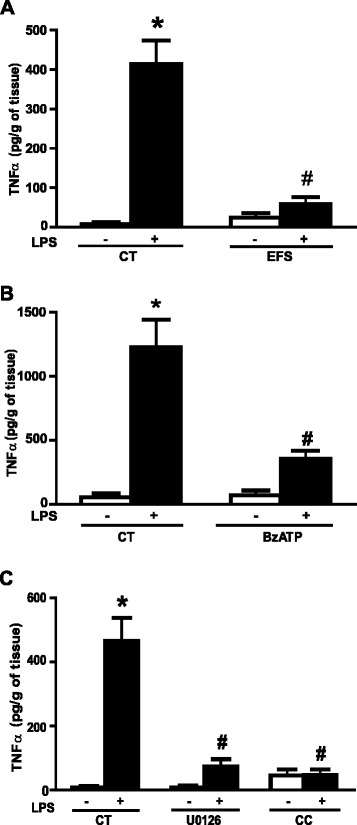
**ENS activation in human LMMP inhibits the LPS-induced TNF-α production.** **(A)** The impact of electrical field stimulation (EFS) on TNF-α secretion by the human LMMP treated (+) or not (−) with LPS (0.1 μg/ml) was performed by ELISA (four independent experiments each performed in triplicate; CT means control). **(B)** The involvement of P2X7 receptor on TNF-α secretion were measured by ELISA in human LMMP pretreated, or not, with BzATP (P2X7 agonist; 100 μM) before LPS stimulation (three independent experiments each performed in triplicate; CT means control). **(C)** ERK- and AMPK-dependent pathway contribution to TNFα production induced by LPS were assessed using MEK1/2 inhibitor (U0126; 10 μM) and AMPK inhibitor (C compound, CC; 10 μM) co-treatment on human LMMP treated, or not, with LPS for seven hours (three independent experiments each performed in triplicate; CT means control). Values represent the mean ± SEM (Mann-Whitney U test; *P <0.05 as compared with control without LPS; #*P* <0.05 LPS with EFS, agonist versus LPS alone). AMPK, 5’-AMP-activated protein kinase; BzATP, 2’(3’)-O-(4-benzoylbenzoyl) adenosine-5'-triphosphate triethylammonium salt; CT, control; CC, C compound; CT, control; EFS, electrical field stimulation; ERK, extracellular signal-regulated kinase; LMMP, longitudinal muscle/myenteric plexus; LPS, lipopolysaccharide; MEK1/2, mitogen-activated protein kinase kinase 1/2; SEM, standard error of the mean; TNF-α, tumor necrosis factor alpha.

To assess if the induction of TNF-α production by LPS and its regulation by EFS also occurs in human samples, we measured LPS-induced TNF-α production in the supernatants of human LMMP after, or not, EFS. EFS had no significant effect on basal TNF-α production. However, EFS significantly inhibited TNF-α production induced by LPS in the LMMP (Figure 4A). The application of BzATP, a P2X7 agonist, induced a similar reduction in TNF-α production (Figure 4B). Pretreatment of LMMP with U0126 or compound C inhibitors of the ERK or AMPK pathways, respectively, significantly reduced TNF-α production (Figure 4C). These results show that in human LMMP as in rENSpc, LPS induces an increase in TNF-α production that depends on AMPK and ERK pathways, and that ENS activation reduces this production by stimulation of the P2X7 receptors.

### Rat enteric nervous system primary cultures upregulate the neuronal expression of TLR2 in response to lipopolysaccharide stimulation

**Figure 5:**
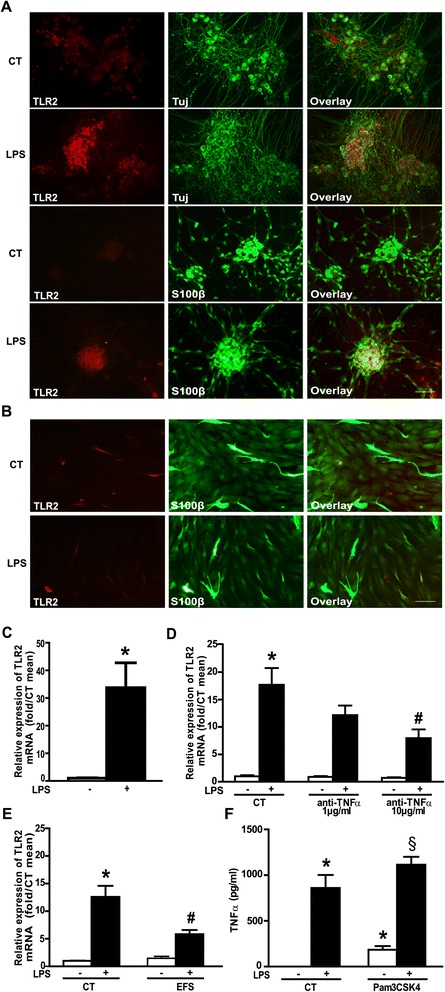
**LPS-induced TNF-α production leads to TLR2 upregulation and TLR2 contributes to TNFα production.** **(A)** Localization of TLR2 on neuronal or glial cells of the rENSpc was analyzed by immunocytochemistry using anti-TLR2, –Tuj (β-tubulin III) and –S100β antibodies on cultures treated for seven hours with LPS (0.1 μg/ml), or not. Scale bar: 50 μm. **(B)** Expression of TLR2 was also observed in human EGC cultures by immunocytochemistry using anti-TLR2 and –S100β antibodies on cultures treated for seven hours with LPS (0.1 μg/ml), or not. **(C)** Relative expression of TLR2 mRNA in rENSpc treated (+), or not (−), with LPS for seven hours (15 independent samples). Values represent the mean ± SEM of TLR2 mRNA quantification against RPS6 (Mann-Whitney U test; *P <0.05 as compared with control without LPS). **(D)** Quantification of TLR2 mRNA in rENSpc treated (+), or not (−), with LPS (0.1 μg/ml) in the presence of, or not, anti-TNF-α (1 μg/ml and 10 μg/ml), was performed by RT-qPCR (five independent samples) (Mann-Whitney U test; *P <0.05 as compared with control without LPS; #*P* <0.05 as compared with LPS treatment without anti-TNFα). **(E)** TLR2 mRNA expression in rENSpc treated (+), or not (−), with LPS in the presence of, or not, EFS stimulation (11 to 16 independent samples). TLR2 mRNA quantification relative to S6 was expressed as induction fold to the control mean (Mann-Whitney U test; *P <0.05 as compared with control without LPS; #*P* <0.05 as compared with LPS treatment without EFS). **(F)** ENS cultures were treated (+), or not (−), with LPS (0.1 μg/ml) and Pam3CSK4 (TLR1/2 agonist; 0.1 μg/ml). Values represent the mean ± SEM of eight independent experiments (Mann-Whitney U test; *P <0.05 as compared with control without LPS; §*P* <0.05 as compared with Pam3CSK4 without LPS).

To further investigate the ENS response to LPS, we measured, using qPCR, *TLR2* mRNA expression in rENSpc. *TLR2* mRNA was detected in control conditions and LPS treatment induced a strong increase in its expression (Figure 5C). To determine if this regulation also occurred at the protein level, and to identify the cells expressing TLR2, we compared immunoreactivity for TLR2 with immunoreactivity for the neuronal marker Tuj or for the glial marker S100β on rENSpc stimulated, or not, with LPS. TLR2 immunoreactivity was detected slightly in our control conditions but clearly appeared in Tuj-positive cells, and rarely in S100β-positive cells, when rENSpc were treated with LPS (Figure 5A). To confirm that TLR2 expression is mainly neuronal, we have analyzed TLR2 immunoreactivity in human EGC. The glial marker S100β staining of cultures of human EGC always present highly positive cells and low positive cells. Only the highly S100β-positive EGC also expressed the TLR2 (Figure 5B), but no changes were observed after LPS stimulation. All together these data show that mainly enteric neurons express the TLR2 and respond to LPS through TLR2 upregulation.

### Lipopolysaccharide-induced TNF-α production participates in TLR2 upregulation and can be prevented by electrical field stimulation activation of the enteric nervous system

To determine if TNF-α production induced by LPS could be involved in the TLR2 upregulation observed in enteric neurons in response to LPS, we measured *TLR2* mRNA in rENSpc stimulated by LPS in the absence or presence of TNF-α-neutralizing antibodies. Anti-TNF-α antibodies significantly reduced the expression of the *TLR2* mRNA induced by LPS (Figure 5D). In the same manner, EFS stimulation of the rENSpc significantly reduced the *TLR2* mRNA expression induced by LPS (Figure 5E). These data show that TNF-α production induced by LPS upregulated the TLR2 receptor expression and depends on ENS activity.

### TLR2 activation induces TNF-α production

TLR2, as well as TLR4, activation in different cell types can induce TNF-α production [21]. To assess the involvement of TLR2 in the ENS response to LPS, we measured TNF-α production in rENSpc stimulated with the TLR1/2 agonist, Pam3CSK4. Pam3CSK4 induced a significant increase in TNF-α production that could be further increased after the addition of LPS (Figure 5F). These results suggest that TLR2 activation can induce TNF-α production by enteric neurons.

### Interleukin-6 production is potentiated by enteric nervous system activation

**Figure 6:**
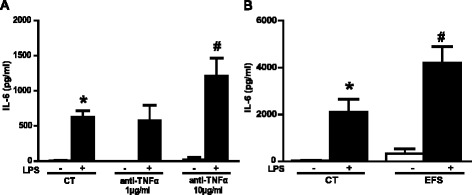
**ENS activation potentiates IL-6 production due to downregulation of TNF-α.** **(A)** LPS-induced IL-6 production in rENSpc in the presence, or not, of anti-TNF-α (1 μg/ml and 10 μg/ml) was measured in an ELISA assay (five to seven independent samples). **(B)** The impact of EFS stimulation on IL-6 production by ENS cultures treated (+) with LPS (0.1 μg/ml) or without (−) (four to eight independent experiments). Values represent the mean ± SEM (Mann-Whitney U test; *P <0.05 as compared with control without LPS; #*P* <0.05 as compared with LPS treatment). EFS, electrical field stimulation; LPS, lipopolysaccharide; rENSpc, rat enteric nervous system; SEM, standard error of the mean; TNF-α, tumor necrosis factor alpha.

An additional functional consequence of TNF-α regulation by ENS activity could be the regulation of other cytokines, such as interleukin-6 (IL-6). To address this issue, we measured IL-6 production by rENSpc treated with LPS in the absence or presence of TNF-α-neutralizing antibodies. Like TNF-α, IL-6 production was increased by LPS, but this was delayed in time compared to the TNF-α response (data not shown). TNF-α-neutralizing antibodies had no effect on basal IL-6 production but, surprisingly, significantly increased that induced by LPS (Figure 6A). EFS stimulation of the ENS increased the IL-6 production (Figure 6B). This suggests that ENS activation, through TNF-α downregulation, can potentiate IL-6 production.

## Discussion

In this study, we showed that stimulation of human or rat ENS by LPS induces neuronal production of TNF-α, which is inhibited by neuronal activity. This regulation partially occurs through the inhibition of the ERK and AMPK neuronal pathways activated by LPS, and can be reproduced by ATP via P2X7 receptors. Furthermore, neuronal activation leads to a TNF-α-dependent inhibition, LPS-induced neuronal TLR2 downregulation, and IL-6 production.

TNF-α production is one of the common inflammatory responses of the gut to LPS endotoxin. An increase in plasma TNF-α level occurs in an animals as early as 90 minutes after an intraperitoneal injection of LPS [22]. In addition to this acute TNF-α production, an elevated level of circulating TNF-α can be measured during chronic inflammation, such as IBD [23]. In this context, our work addressed the question of identifying the ENS as TNF-α-producing cells, among other known cells of the gut. Indeed, in the gut, TNF-α is produced by activated macrophages, CD4+ lymphocytes, natural killer [24] and dendritic cells. In addition, nonimmune cells such as epithelial cells, endothelial cells, smooth muscle cells, vascular smooth muscle cells, fibroblasts and cardiac myocytes have also been shown to produce TNF-α [25,26]. Our work showed that enteric neurons in primary culture produce TNF-α in response to LPS stimulation. In contrast, we were unable to detect any TNF-α production, with or without LPS, in EGCs (from rENSpc, or from rat or human EGCs). Besides producing TNF-α, enteric neurons have also previously been shown to produce other cytokines and chemokines, such as IL-8 [27]. Production of TNF-α has also been shown in the CNS, by neurons [28,29] and also, in contrast to the ENS, by astrocytes [19,30]. Altogether, our study suggests that enteric neurons could, as resident cells, relay a first inflammatory reaction to LPS by producing TNF-α.

It is well known that the synthesis of biologically active TNF-α is regulated at several different levels and that mitogen-activated protein kinase (MAPK) pathways are critical for its production, as well as for that of several other proinflammatory cytokines [31]. LPS can signal through all three p38, c-Jun N-terminal kinase (JNK) and ERK MAPKs [32,33], and through different downstream effectors to regulate *TNF-α* transcription [34], as well as *TNF-α* translation [35,36]. We observed that ERKs were activated in enteric neurons after LPS challenge, and that ERK pathway inhibition significantly, but only partially, inhibited TNF-α production and transcription. It was more surprising to observe the basal activation of the AMPK pathway in enteric neurons and the drastic inhibition of LPS-induced TNF-α transcription and production by the AMPK inhibitor, C compound. Indeed, several studies, using various inflammatory stimuli, have shown that the energy-sensing enzyme AMPK has anti-inflammatory actions thought to be largely mediated by negative regulation of the nuclear factor-kappa B (NF-κB) pathway [37-41]. However, some evidence emphasizes the dual effect of C compound on the inflammatory response, which upregulated the release of TNF-α production in non-stimulated microglia, but downregulated TNF-α release in LPS-stimulated microglia [42]. This complex regulation is consistent with the inhibition of ERK and AMPK phosphorylation by ENS activation.

In a more general consideration, ERK is known to be activated in response to stimuli linked to neuronal activity [43]. However, it is also well known that the activation of MAPK is transient [43,44] and hardly ever sustained in physiological conditions, due to desensitizing or negative feedback processes. We could assume that ENS activation precociously activates ERK pathways that might not be further impacted by LPS stimulation. We also observed that EFS effects on LPS-induced TNF-α production can be reproduced with ATP (Figure 2D), but not with acetylcholine, serotonin or vasoactive intestinal polypeptide (unpublished results). The ATP-induced inhibition of TNF-α production was reproduced by activation of the purinergic receptor, P2X7, and blocked in part by a P2X7 antagonist. Various studies have recently reported a wide range of actions of P2X7 in enteric neurons. First, P2X7 expression has been reported to be widely expressed in enteric neurons [45,46]. Activation of P2X7 has been shown to increase neuronal excitability [45], as well as being responsible for neuronal death induced by inflammation [47]. Here, we observed that activation of the P2X7 receptor could limit TNF-α production in enteric neurons.

TNF-α can have various effects on different cell types, such as immune cells, intestinal epithelial cells or even muscle cells during intestinal inflammation [48-52]. Our study suggests that production of TNF-α by enteric neurons can modulate neuronal functions in an autocrine manner, as demonstrated by the TNF-α-dependent increase in neuronal TLR2 expression. TNF-α has also been reported to have multiple other effects on enteric neurons. For instance, TNF-α has been shown to inhibit norepinephrine release from the rat myenteric plexus [53], to modulate neuronal excitability [54] and even have neuroprotective effects [55]. TNF-α production by enteric neurons could also induce enteric glial reactivity [56]. In addition, we have shown that TNF-α limits LPS-induced IL-6 production, which is, however, amplified when the ENS is activated. This pleiotropic cytokine has a central role in generating acute-phase responses, inflammation and lymphocyte differentiation. Concerning the ENS, IL-6 increases neuronal excitability [57-59], and can participate in gastrointestinal dysfunction associated with intestinal inflammation or irritable bowel syndrome. In addition, IL-6 can also reduce enteric neuronal survival [60]. In our study, the cellular origin of IL-6 was not investigated but may involve both EGCs and intestinal smooth muscle cells, both of which respond to TNF-α stimulation by IL-6 production [61,62].

We also demonstrated that LPS-induced TNF-α production by enteric neurons induced the neuronal expression of TLR2. TLR2 expression induced by LPS has already been described in endothelial cells [63], and our work extends this regulation to neuronal cells, and shows that this regulation occurs through TNF-α production. TLR2 is a TLR of particular interest because it mediates reaction to inflammation [64], could have nonimmune functions and effects opposite those mediated by TLR4 [65] and its expression is induced in the intestines of patients with IBD [66]. The LPS-induced increase in TLR-2 expression in enteric neurons could participate in a protective response to inflammatory or infectious challenges, as recent data have reported that genetic deficiency of TLR2 induces anomalies in the structure, neurochemical coding and function of ENS. This is in part due to glial cell-derived neurotrophic factor (GDNF) production [14], which has been shown to have central neuroprotective and neuromodulatory effects in the ENS [67,68].

## Conclusions

Although *in vivo* data are lacking to make conclusions about the impact of ENS activity on the intestinal response to LPS, our study is the first demonstration that the ENS can produce TNF-α, that its activation inhibits TNF-α production and TLR2 expression, and also potentiates IL-6 production. This study thus defines the ENS as a potent player in neuroimmune inflammation.

## Additional files

Additional file 1:
**EFS induces only neuronal activation.**
Additional file 2:
**Expression of TLR4 in ENS and EGC cultures is not modified by LPS treatment.**

## Abbreviations

ACC: Acetyl-coenzyme A Carboxylase
AMPK: 5’-Adenosine monophosphate-activated protein kinase
ATP: Adenosine-5'-triphosphate
BzATP: 2’(3’)-O-(4-benzoylbenzoyl) adenosine-5'-triphosphate triethylammonium salt
CC: C compound
CT: Control
EFS: Electrical field stimulation
EGC: Enteric glial cell
ENS: Enteric nervous system
ERK: Extracellular signal-regulated kinase
IBD: Inflammatory bowel disease
IL-6: Interleukin-6
LMMP: Longitudinal muscle/myenteric plexus
LPS: Lipopolysaccharide
MAPK: Mitogen-activated protein kinase
MEK: Mitogen-activated protein kinase kinase
mRNA: messenger ribonucleic acid
qPCR: Quantitative polymerase chain reaction
rENSpc: Rat enteric nervous system primary culture
RPS6: Ribosomal S6 protein
S100β: S100 calcium binding protein beta
SEM: Standard Error of the Mean
SMA: Smooth Muscle Actin
TLR: Toll-like receptor
TNFα: Tumor necrosis factor alpha
Tuj: βIII-tubulin
