# Supplementary material for: Modulation of lipopolysaccharide-induced neuronal response by activation of the enteric nervous system
Source: J Neuroinflammation. 2014 Dec 12;11:202. doi: 10.1186/s12974-014-0202-7 (PMC4279994; doi:10.1186/s12974-014-0202-7)
Supplement: Additional file 1: — EFS induces only neuronal activation. [file 12974_2014_202_MOESM1_ESM.pdf]

Additional file 1

EFS induces only neuronal activation.

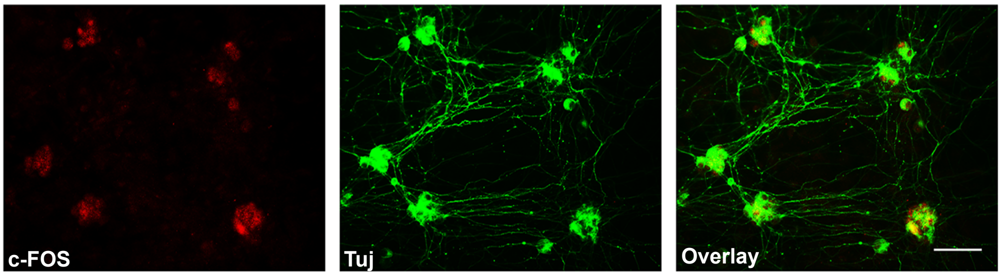

Immunocytochemical double labeling of rENSpc were performed using anti-c-Fos (Calbiochem) and anti-Tuj (βIII-tubulin; Sigma) antibodies after 7 hours of EFS. Scale bar : 50μm.
