# Supplementary material for: Modulation of lipopolysaccharide-induced neuronal response by activation of the enteric nervous system
Source: J Neuroinflammation. 2014 Dec 12;11:202. doi: 10.1186/s12974-014-0202-7 (PMC4279994; doi:10.1186/s12974-014-0202-7)
Supplement: Additional file 2: — Expression of TLR4 in ENS and EGC cultures is not modified by LPS treatment. [file 12974_2014_202_MOESM2_ESM.pdf]

Additional file 2

Expression of TLR4 in ENS and EGC cultures is not modified by LPS treatment.

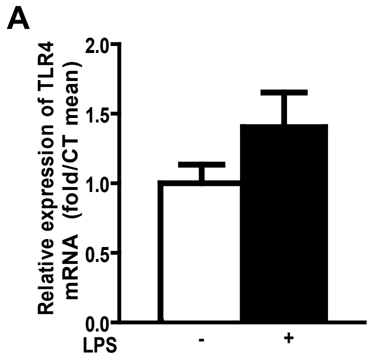

Relative expression of TLR4 mRNA in rENSpc treated (+) or not (-) by LPS for 7 hours.

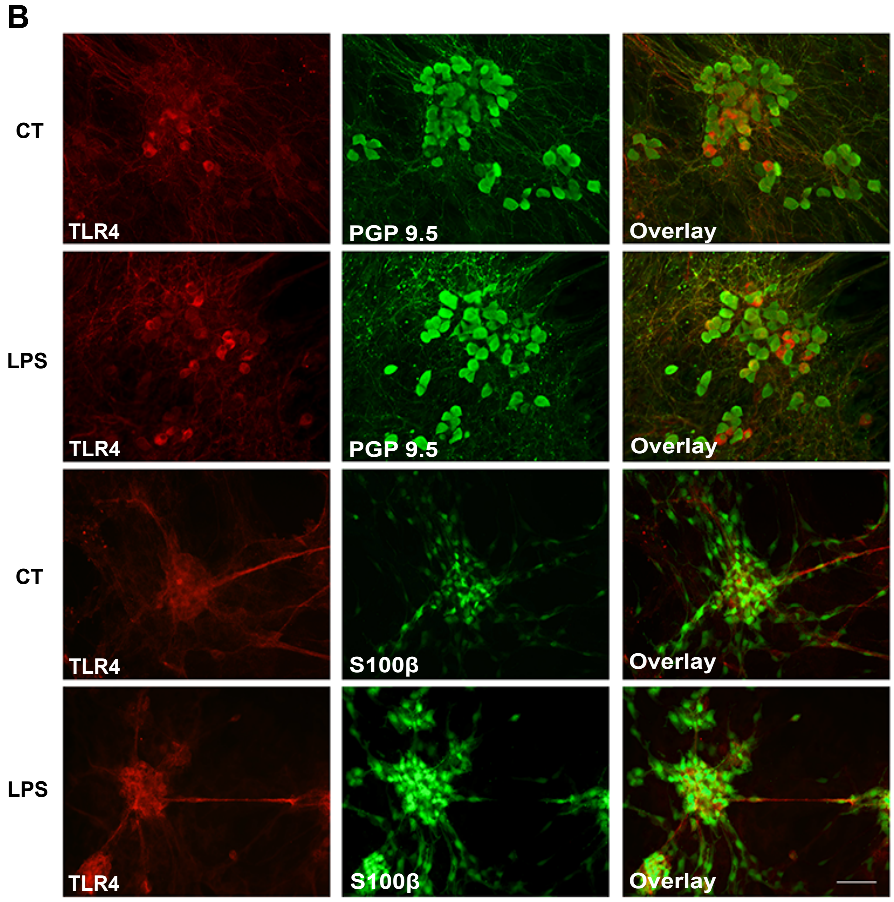

Localization of TLR4 on neuronal and glial cells in rENSpc was analyzed by immunocytochemistry using anti-TLR4 (Imgenex), anti-PGP 9.5 (Ultraclone Limited) and anti-S100β (DakoCytomation) antibodies. Scale bar : 50µm.

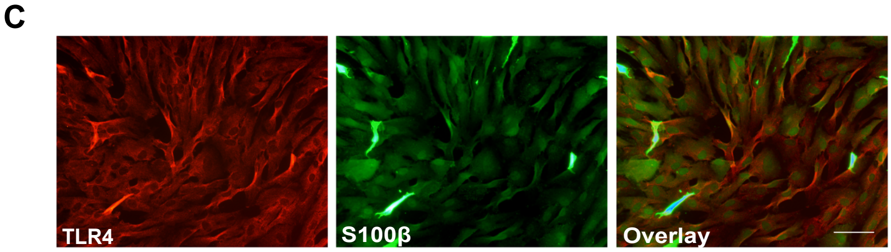

Expression of TLR4 on human enteric glial cells in EGC primary cultures was analyzed by immunocytochemistry using anti-TLR4 and anti-S100β antibodies. Scale bar : 50µm.
